# Effect of acupuncture for non-motor symptoms in patients with Parkinson’s disease: A systematic review and meta-analysis

**DOI:** 10.3389/fnagi.2022.995850

**Published:** 2022-10-06

**Authors:** Qinglian Li, Chunxiao Wu, Xiaoling Wang, Zhen Li, Xiaoqian Hao, Lijun Zhao, Mengzhu Li, Meiling Zhu

**Affiliations:** ^1^Shenzhen Hospital of Integrated Traditional Chinese and Western Medicine, Guangzhou University of Chinese Medicine, Shenzhen, China; ^2^The Research Center of Basic Integrative Medicine, Guangzhou University of Chinese Medicine, Guangzhou, China

**Keywords:** Parkinson’s disease, non-motor symptoms, acupuncture, meta-analysis, randomized controlled trial

## Abstract

**Background:**

Although non-motor symptoms of Parkinson’s disease (PD) are serious, effective treatments are still lacking. Acupuncture may have clinical benefits for non-motor symptoms of PD patients, but high-quality evidence supporting this possibility is still limited. Hence, we conducted this meta-analysis to evaluate the effect of acupuncture treatment on non-motor symptoms in patients with PD.

**Methods:**

Randomized controlled trials (RCTs) of acupuncture treatment for PD were retrieved from the following electronic databases: Medline (OVID), Embase (OVID), Cochrane Library, Web of Science, China National Knowledge Infrastructure, Chinese BioMedical Literature Database, Chonqing VIP (CQVIP), and Wangfang database. Studies evaluating non-motor symptoms of PD were retrieved. Methodological quality was assessed using the Cochrane Handbook for Systematic Reviews of Interventions.

**Results:**

A total of 27 RCTs were included, among which 8 outcomes related to non-motor symptoms were evaluated. The results showed that acupuncture combined with medication had benefits for PD-related insomnia relative to medication alone or sham acupuncture [standardized mean difference (SMD) = 0.517; 95% confidence interval (CI) = 0.242–0.793; *p* = 0.000], and acupuncture treatment had benefits at 8 weeks (SMD = 0.519; 95% CI = 0.181–0.857; *p* = 0.003). Regarding depression, acupuncture treatment was more effective (SMD = −0.353; 95% CI = −0.669 to −0.037; *p* = 0.029) within 2 months (SMD = −0.671; 95% CI = −1.332 to −0.011; *p* = 0.046). Regarding cognition, quality of life, and Unified Parkinson’s Disease Rating Scale (UPDRS) I and II scores, acupuncture treatment was effective [SMD = 0.878, 95% CI = 0.046–1.711, *p* = 0.039; SMD = −0.690, 95% CI = −1.226 to −0.155, *p* = 0.011; weighted mean difference (WMD) = −1.536, 95% CI = −2.201 to −0.871, *p* = 0.000; WMD = −2.071, 95% CI = −3.792 to −0.351, *p* = 0.018; respectively]. A significant difference was not found in terms of PD-related constipation. Only one study evaluated PD-related fatigue.

**Conclusion:**

The results of the analysis suggested that acupuncture treatment could ameliorate the symptoms of depression, quality of life, cognition, total mentation, behavior and mood, and activities of daily living in PD patients. Nevertheless, more prospective, well-designed RCTs with larger sample sizes are required to confirm our findings.

## Introduction

Parkinson’s disease (PD) is a neurodegenerative disorder characterized by a myriad of motor symptoms and non-motor symptoms affecting approximately 6.1 million individuals worldwide in 2016, 2.4 times higher than in 1990 (GBD 2016 Neurology Collaborators, 2019). The duration of symptoms can span decades, and the accumulating disability can lower quality of life. For society, PD has caused a global burden, especially in terms of deaths and disability, which has been rapidly rising in the past two decades (Deuschl et al., 2020). Therapies such as dopamine agonists and dopaminergic medications are the most effective therapies for PD but have a limited effect on non-motor symptoms (Kim et al., 2009; Connolly and Lang, 2014; Armstrong and Okun, 2020). In addition, non-motor symptoms have often been neglected in clinical practice, although some researchers in the last decade have called for more attention because they are the main key to quality of life of patients (Poewe, 2010; Asakawa et al., 2016; Chaudhuri and Sauerbier, 2016). Some non-motor symptoms of PD patients precede the motor symptoms by several years, are present early in the disease course and could be the drivers of significant disability as the disease progresses (Haehner et al., 2007; Seppi et al., 2019; Gilbert and Khemani, 2022). Moreover, some researchers have emphasized the disparities between the need for treatment for non-motor symptoms and the lack of clinical trial data evaluating these symptoms (Zesiewicz et al., 2010). Although the number of convincing studies have been increasing in recent years, evidence-based treatments remain limited (Seppi et al., 2019). Therefore, it is important to prioritize the development of an analysis of the management of non-motor symptoms.

Non-motor symptoms of PD include symptoms such as constipation, fatigue, cognitive impairment, and sleep and affective disturbances. Symptomatic therapies for non-motor symptoms of PD are similar to those for general populations (those without PD), while evidence for the efficacy of these therapies, specifically in individuals affected by PD varies (Armstrong and Okun, 2020). Multidisciplinary approaches and non-pharmacologic treatment strategies have been encouraged (Goldman and Guerra, 2020; Bloem et al., 2021). Acupuncture as a non-invasive procedure and an alternative therapy has received increasing attention in recent years. Accumulating studies investigating the effectiveness of acupuncture targeting PD or other disorders, such as depression, insomnia, cognitive impairment and so on, have had some positive results (Lee and Lim, 2017; Goldman and Guerra, 2020; Yeung et al., 2021). However, high-quality evidence supporting significant effects of acupuncture on non-motor symptoms of PD is lacking. Therefore, we carried out a meta-analysis of randomized controlled trials (RCTs) to reach a solid conclusion with a larger sample size. We aimed to determine the effects of acupuncture on the non-motor symptoms of PD and provide evidence-based recommendations for PD patients with non-motor symptoms.

## Materials and methods

This meta-analysis (registration No. CRD42022295938) focused on RCTs involving acupuncture and acupuncture combined with medication interventions on non-motor symptoms in PD patients, which abided by the Preferred Reporting Items for Systematic Reviews and Meta-Analyses (PRISMA) statement (Page et al., 2021).

### Search strategy

We searched Medline (OVID), Embase (OVID), the Cochrane Library, Web of Science, China National Knowledge Infrastructure (CNKI), Chinese BioMedical Literature Database (CBM), Chonqing VIP (CQVIP), and Wangfang database from their inception through November 2021. Databases were searched using three main components: disease (PD), intervention (acupuncture), and study type (randomized clinical trial). The complete search strategy is shown in Supplementary Appendix.

### Inclusion criteria/exclusion criteria

Articles meeting the following criteria were included: (1) population: patients diagnosed as PD using any recognized diagnostic criteria (UK Parkinson’s Disease Society Brain Bank Criteria for the diagnosis of PD) (Hughes et al., 2001), MDS clinical diagnostic criteria for PD (Postuma et al., 2015), diagnostic criteria for PD in China (2016) (Parkinson’s Disease and Movement Disorders Group of Neurology Branch of Chinese Medical Association, 2016); (2) intervention: acupuncture treatment including acupuncture alone or acupuncture combined with conventional treatment; (3) comparators: no treatment, sham acupuncture, sham acupuncture combined with conventional treatment or conventional treatment alone (conventional treatment included Antiparkinson drug and other drugs for the non-motor symptoms of PD); (4) outcome measure: involving some metric related to non-motor symptoms, for instance: the primary outcomes of this meta-analysis were insomnia [assessed by the Pittsburgh Sleep Quality Index (PSQI) (Buysse et al., 1989) and Parkinson’s Disease Sleep Scale (PDSS) (Chaudhuri et al., 2002)], depression [assessed by the Hamilton Depression Scale (HAMD) (Hamilton, 1980) and Hospital Anxiety Depression Scale (HADS, depression)] (Zigmond and Snaith, 1983), cognition [measured by the Montreal Cognitive Assessment (MoCA)] (Nasreddine et al., 2005), constipation and fatigue as non-motor outcomes. Unified Parkinson’s Disease Rating Scale (UPDRS) I and II scores (Ramaker et al., 2002), evaluating behavioral and emotional problems and activities of daily living, respectively, were used as secondary outcome indices. Quality of life [the Parkinson’s Disease Questionnaire (PDQ-39)] was also assessed and included as a secondary outcome index; (5) study design: randomized control trials. And language was not restricted.

Studies were excluded if they reported only the response rate or did not have data available for effect size estimates. Duplicate or secondary publications on the same sample were excluded. Conference abstracts, protocols or presentations were also excluded. Two authors (XW and ML) screened the articles on the basis of titles and abstracts. If they could not determine eligibility of a study through this procedure, they selected the article for a subsequent full-text assessment.

### Data extraction

Two reviewers (LZ and ZL) extracted and checked the data, and disagreements were resolved by a third-party expert (C W). Clinical characteristics (population, interventions, comparators, and outcome measures), details of the interventions and comparators, time points of measurement and the results before and after the treatments were extracted from each article.

### Quality assessment

Two reviewers (QL and XH) independently assessed the quality of the included trials using the revised Cochrane risk of bias, version 2 (RoB 2) tool (Sterne et al., 2019). Disagreements were settled by discussion with another reviewer, CW. Each trial was assessed as having a low or high or some concerns of bias in terms of randomization process, deviations from intended interventions, missing outcome data, measurement of the outcome, selection of the reported result.

### Statistical analysis

Data were synthesized and analyzed through STATA (version 13). We calculated *I*^2^ statistics and *Q* statistics to determine which effect model was appropriate (Higgins et al., 2003). If the *p*-value was ≤0.05 or *I*^2^ was >40%, this was taken as an indicator of notable heterogeneity among the trials, and a random effect model was chosen (Deeks et al., 2022). The sources of heterogeneity were further explored using subgroup analyses. If the *p*-value was greater than 0.05 or *I*^2^ was no more than 40%, indicating that heterogeneity was low, a fixed effects model was applied. The weighted mean difference (WMD) or standardized mean difference (SMD) with 95% confidence intervals (CIs) was calculated to analyze continuous outcomes. If the assessment tool was the same, WMD was applied to analyze continuous data; otherwise, SMD was used. If necessary, sensitivity analyses were performed to test the stability of the results. Moreover, the risk of publication bias was evaluated by funnel plots or Egger’s test.

## Results

### Study identification and selection

**FIGURE 1:**
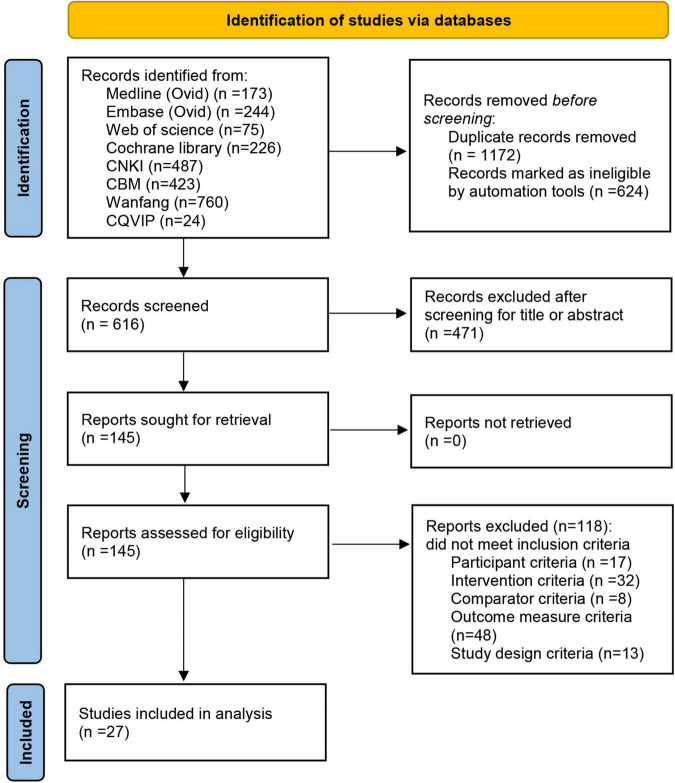
Flowchart.

In total, 2,412 potentially eligible articles were identified through database searches, from which 1,172 duplicate articles were removed. After screening the titles and abstracts, we selected 145 articles for full-text dual review after excluding 1,095 articles on the basis of the inclusion criteria. A total of 118 of 145 articles were excluded, and the detailed reasons are shown in the flow diagram (Figure 1). Finally, 27 articles were included in this meta-analysis (Yang, 2009; Cho et al., 2012, 2018; Xia et al., 2013; Liang, 2014; Chen, 2015; Toosizadeh et al., 2015; Wang et al., 2015, 2019; Kluger et al., 2016; Li, 2016; Tian et al., 2016; Zhou et al., 2016; Aroxa et al., 2017; Dong et al., 2018; Li L. et al., 2018; Li Z. et al., 2018; Liu et al., 2018; Luo and Zhang, 2019; Zhan and Wang, 2019; Li et al., 2020, 2021; Wu, 2020; Xu et al., 2020; Yuan et al., 2020; Zhang, 2020; Wu et al., 2021). The specific screening procedure is summarized in Figure 1.

### Study characteristics

Among the 27 eligible articles, 9 studies focused on insomnia (Liang, 2014; Wang et al., 2015; Kluger et al., 2016; Zhou et al., 2016; Aroxa et al., 2017; Dong et al., 2018; Wu, 2020; Xu et al., 2020; Li et al., 2021), 9 studies focused on depression (Cho et al., 2012, 2018; Xia et al., 2013; Wang et al., 2015; Kluger et al., 2016; Tian et al., 2016; Xu et al., 2020; Yuan et al., 2020; Li et al., 2021), 5 studies focused on cognition (Liang, 2014; Wang et al., 2015, 2019; Kluger et al., 2016; Wu et al., 2021), 4 studies focused on constipation (Li L. et al., 2018; Luo and Zhang, 2019; Zhan and Wang, 2019; Zhang, 2020), one study focused on fatigue (Kluger et al., 2016), 9 studies focused on quality of life (Yang, 2009; Cho et al., 2012, 2018; Liang, 2014; Chen, 2015; Kluger et al., 2016; Li, 2016; Liu et al., 2018; Li et al., 2020), 9 studies focused on UPDRS I scores (Yang, 2009; Cho et al., 2012; Chen, 2015; Toosizadeh et al., 2015; Li L. et al., 2018; Luo and Zhang, 2019; Wang et al., 2019; Xu et al., 2020; Yuan et al., 2020) focused on UPDRS I, and 12 studies focused on UPDRS II scores (Yang, 2009; Cho et al., 2012, 2018; Liang, 2014; Chen, 2015; Toosizadeh et al., 2015; Li, 2016; Li L. et al., 2018; Li Z. et al., 2018; Luo and Zhang, 2019; Wang et al., 2019; Xu et al., 2020). A total of 23 studies used a 2-group parallel design, 3 of which compared real acupuncture with sham acupuncture, 7 of which compared acupuncture with conventional treatments, and 13 of which compared the combination of acupuncture and conventional treatments (such as Madopar or Levodopa) with conventional treatments. A total of four studies used a three-arm study design. The parameters of electroacupuncture involved a continuous wave and dilatational wave, and the frequency varied from 1.5 to 100 Hz. The acupuncture intervention duration ranged from 15 to 40 min per session, with one to seven sessions per week. The intervention courses ranged from 4 to 24 weeks. A total of 2 studies were completed in America, 2 in Korea, 1 in Brazil, and the remaining 22 in China. The details of the study characteristics are exhibited in Table 1.

**TABLE 1:** Basic characteristics.

References	Study population	Intervention	Control 1	Control 2	Outcomes measured	Measurement time points
Li L. et al., 2018	PD H-Y stage 1–3	Electroacupuncture Freq: dilatational wave 2/15 Hz, once/d Duration: 30 min Course: 4 w	Polyethylene glycol		UPDRS I, UPDRS II, anal rectum dynamics	4 w
Luo and Zhang, 2019	PD (H-Y stage not mentioned)	Electroacupuncture Freq: continuous wave 1.5 Hz, once/d Duration: 20 min Course: 4 w	Phenolphthalein tablets		UPDRS I, UPDRS II, anal rectum dynamics	4 w
Zhan and Wang, 2019	PD (H-Y stage not mentioned)	Acupuncture Freq: once/d, 6 d/w Duration: 40 min Course: 8 w	Maren Wan		BSFS, PAC-QOL	8 w
Zhang, 2020	PD (H-Y stage not mentioned)	Acupuncture Freq: 3 times/w Duration: 30 min Course: 4 w	Maren soft capsules		PAC-QOL, Bristol	4 w
Aroxa et al., 2017	PD H-Y stage 1–3	Acupuncture + Antiparkinson drug Freq: 8 times/w Duration: 30 min Course: 8 w	Antiparkinson drug		PDSS, MMSE	8 w
Wang et al., 2015	PD (H-Y stage not mentioned)	Acupuncture + Antiparkinson drug Freq: 100 Hz, 3 d/time Duration: 30 min Course: 2 m	Antiparkinson drug		PSQI, HAMD, MMSE, MoCA,	2 m
Xu et al., 2020	PD H-Y stage 1–4	Electroacupuncture + Levodopa and Benserazide Freq: continuous waves 100 Hz, 4 times/week Duration: 30 min Course: 8 w	Levodopa and Benserazide		UPDRS I, UPDRS II, PDSS, SDS	4, 8, 12 w
Dong et al., 2018	PD H-Y stage 1–3	Electroacupuncture Freq: continuous waves 2 Hz, once/day Duration: 30 min Course: 30 d	Madopar		PDSS	30 d
Li et al., 2021	PD (H-Y stage not mentioned)	Electroacupuncture + Madopar Freq: dilatational wave 2/15 Hz, once/d Duration: 30 min Course: 8 w	Madopar		PDSS, PSQI, SDS, UPDRS	8 w
Wu, 2020	PD H-Y stage 1.5–4	Acupuncture Freq: once/day, 6 times/week Duration: 40 min Course: 4 w	Estazolam		PDSS, PSQI	4 w
Zhou et al., 2016	PD (H-Y stage not mentioned)	Electroacupuncture + Madopar Freq: continuous waves 2 Hz, once/d Duration: 20 min Course: 8 w	Madopar		PDSS, UPDRS II	8 w
Kluger et al., 2016	PD (H-Y stage not mentioned)	Acupuncture Freq: twice/week Duration: 30 min Course: 6 w	Sham acupuncture		MFIS, HADS depression, PDSS, PDQ-39	6 w
Cho et al., 2018	PD H-Y stage 1–4	Acupuncture Freq: twice/week Duration: 15 min Course: 12 w	Sham acupuncture	Antiparkinson drug	BDI, PDQL, UPDRS II	12 w
Chen, 2015	PD H-Y stage 1–5	Acupuncture + Madopar Freq: 2 consecutive d, followed by a 2-d rest Duration: 30 min Course: 24 w	Madopar		UPDRS I, UPDRS II	24 w
Li et al., 2020	PD H-Y stage 1–4	Electroacupuncture + Antiparkinson drug Freq: 10/50 Hz, 3 times/w Duration: 30 min Course: 12 w	Antiparkinson drug		PDQ-39	8, 12 w
Li, 2016	PD H-Y stage 1–3	Acupuncture + Antiparkinson drug Freq: 2 times/w Duration: 30 min Course: 12 w	Antiparkinson drug	Sham acupuncture + Antiparkinson drug	UPDRS II, PDQ-39	4, 8, 12 w
Liang, 2014	PD H-Y stage 1–3	Acupuncture Freq: every other day, 3 times/w Duration: 30 min Course: 6 m	Madopar		PDQ-39, PDSS, UPDRS II	1, 3, 6 m
Liu et al., 2018	PD H-Y stage 1–3	Electroacupuncture + compound Carbidopa tablet Freq: continuous waves, 1.5 Hz, once/d Duration: 20 min Course: 3 m	Compound Carbidopa tablet		PDQ-39	3 m
Yang, 2009	PD (H-Y stage not mentioned)	Acupuncture Freq: 5 times/w Duration: 30 min Course: 4 w	Madopar		UPDRS I, UPDRS II	4 w
Cho et al., 2012	PD (H-Y stage not mentioned)	Acupuncture Freq: twice/w Duration: 20 min Course: 8 w	Not treatment		UPDRS I, UPDRS II, PDQL, BDI	8 w
Tian et al., 2016	PD (H-Y stage not mentioned)	Electroacupuncture + Madopar and Fluoxetine Freq: dilatational wave, every other 2 d Duration: 30 min Course: 3 m	Madopar and Fluoxetine		HAMD17	3 m
Xia et al., 2013	PD (H-Y stage not mentioned)	Electroacupuncture + Madopar and Fluoxetine Freq: dilatational wave, every other day Duration: 30 min Course: 3 m	Madopar and Fluoxetine		HAMD17	3 m
Yuan et al., 2020	PD (H-Y stage not mentioned)	Acupuncture Freq: once/d, every other day Duration: 30 min Course: 3 m	Escitalopram Oxalate		HAMD17, UPDRS I	3 m
Toosizadeh et al., 2015	PD (H-Y stage not mentioned)	Electroacupuncture Freq: 4/100 Hz, once/w Duration: 30 min Course: 3 w	Sham acupuncture		UPDRS I, UPDRS II	3 w
Wang et al., 2019	PD (H-Y stage not mentioned)	Acupuncture + Madopar Freq: once/d Duration: 30 min Course: 30 days	Acupuncture	Madopar	UPDRS I, UPDRS II, MMSE, MoCA	30 d
Li Z. et al., 2018	PD H-Y stage 1–3.5	Acupuncture + Levodopa Duration: 30 min Course: 12 w	Sham acupuncture + Levodopa	Levodopa	UPDRS II	12 w
Wu et al., 2021	PD H-Y stage 2–3	Acupuncture + Madopar Freq: once/d, 5 times/w Duration: 30 min Course: 12 w	Madopar		MoCA	12 w

BDI, Beck Depression Rating Scale; BSFS, Bristol Stool Form Scale; d, day; m, months; H-Y, Hoehn–Yahr; MFIS, Modified Fatigue Impact Scale; min, minute; MMSE, Mini-Mental State Examination; PAC-QOL, Patient Assessment of Constipation Quality of Life Questionnaire; PDQL, Parkinson’s Disease Quality of Life Questionnaire; SDS, Self-Rating Depression Scale; w, week.

### Quality assessment of included studies

**FIGURE 2:**
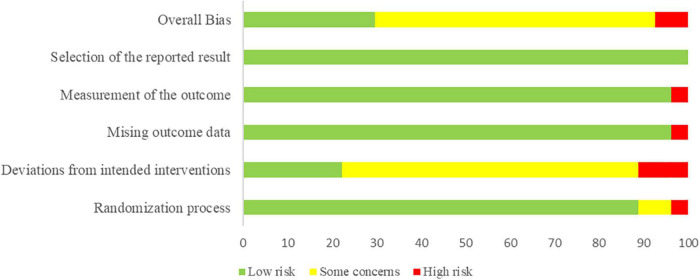
Risk of bias graph.

Risk of bias, version 2 indicated low risk for 5 studies (Kluger et al., 2016; Li, 2016; Li Z. et al., 2018; Luo and Zhang, 2019; Zhang, 2020), high risk for 5 studies (Cho et al., 2012; Liang, 2014; Wang et al., 2015; Li et al., 2020; Wu, 2020), and some concerns for 17 studies (Yang, 2009; Xia et al., 2013; Chen, 2015; Toosizadeh et al., 2015; Tian et al., 2016; Zhou et al., 2016; Aroxa et al., 2017; Cho et al., 2018; Dong et al., 2018; Li L. et al., 2018; Liu et al., 2018; Wang et al., 2019; Zhan and Wang, 2019; Xu et al., 2020; Yuan et al., 2020; Li et al., 2021; Wu et al., 2021). Twenty-one studies reported random sequence generation using proper approaches such as a random number table, computer-generated random numbers, or central stochastic distribution, while the remaining 6 studies did not interpret this procedure. Allocation concealment was performed using opaque, sealed envelopes or was sequentially numbered in 10 studies, whereas 17 studies did not report a specific approach for how allocation concealment was conducted. Overall, 19 studies were judged as some concerns risk of bias in deviations from intended interventions domain because most studies failed to blind the participants and personnel, and only 5 studies reported blinding. Only one study was judged as high risk of bias in missing outcome data domain because some participants were lost to follow-up. Besides, one study was judged as high risk of bias in measurement of the outcome. All studies were judged as low risk of bias in selection of the reported result. Figure 2 provided explicit details methodological quality assessment.

### Primary outcomes

#### Insomnia

**FIGURE 3:**
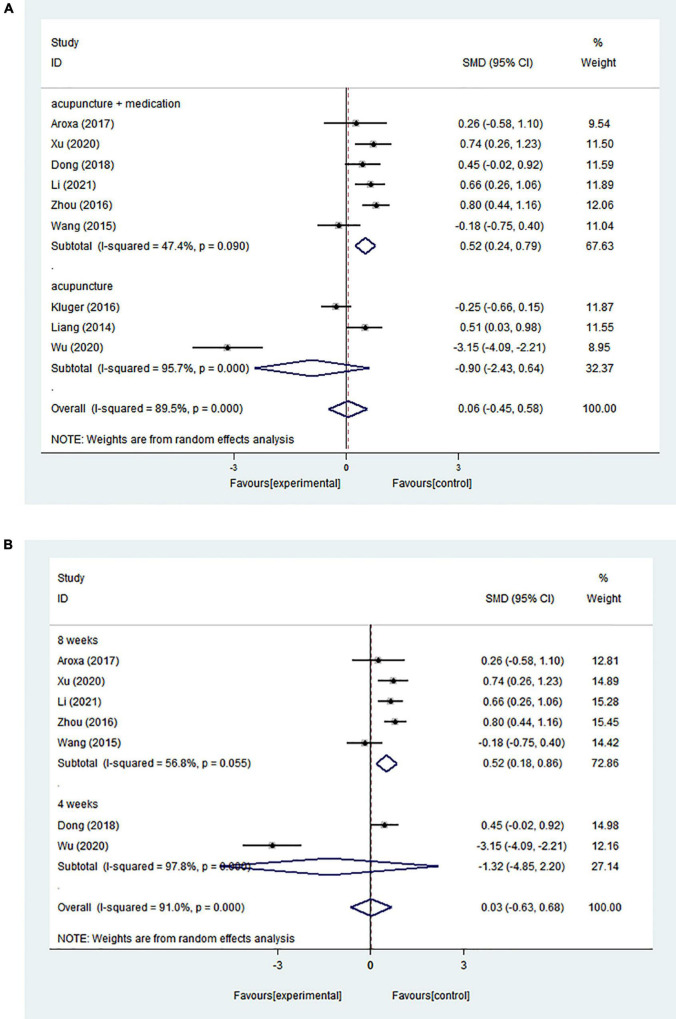
**(A)** Forest plot of effects of acupuncture treatment on PD-related insomnia according to intervention types. **(B)** Forest plot of effects of acupuncture treatment on PD-related insomnia according to course.

A random effects model was used because of the high statistical heterogeneity of the pooled results (*I*^2^ = 89.5%). Nine RCTs showed no significant intervention effect (SMD = 0.064; 95% CI = −0.447 to 0.576; *p* = 0.805) (Figure 3A).

Because of the high heterogeneity of the pooled results, we conducted subgroup analyses to explore sources of heterogeneity. Based on different interventions, studies were separated into those evaluating acupuncture combined with medication and those evaluating acupuncture alone. Six studies assessed the effect of acupuncture combined with medication and showed that acupuncture combined with medication could significantly improve insomnia compared with the control group (medication alone) (SMD = 0.517; 95% CI = 0.242–0.793; *p* = 0.000) (*I*^2^ = 47.4%). Three studies examined the effect of acupuncture alone and demonstrated that there was no significant difference between the acupuncture group and the control group (medication alone or sham acupuncture) (SMD = −0.898; 95% CI = −2.432 to 0.636; *p* = 0.251) (*I*^2^ = 95.7%) (Figure 3A). It was clear that heterogeneity was influenced by intervention type, which may be one of the sources of heterogeneity.

The studies included varying courses of treatment, with five trials assessing insomnia at 8 weeks, and two trials assessing insomnia at 4 weeks. The effect of the intervention treatment for improving PD-related insomnia at 4 weeks was similar to that of the control treatment (SMD = −1.325; 95% CI = −4.853 to 2.204; *p* = 0.462) (*I*^2^ = 97.8%). However, there was a significant difference when the treatment occurred over 8 weeks (SMD = 0.519; 95% CI = 0.181–0.857; *p* = 0.003) (*I*^2^ = 56.8%) (Figure 3B).

#### Depression

**FIGURE 4:**
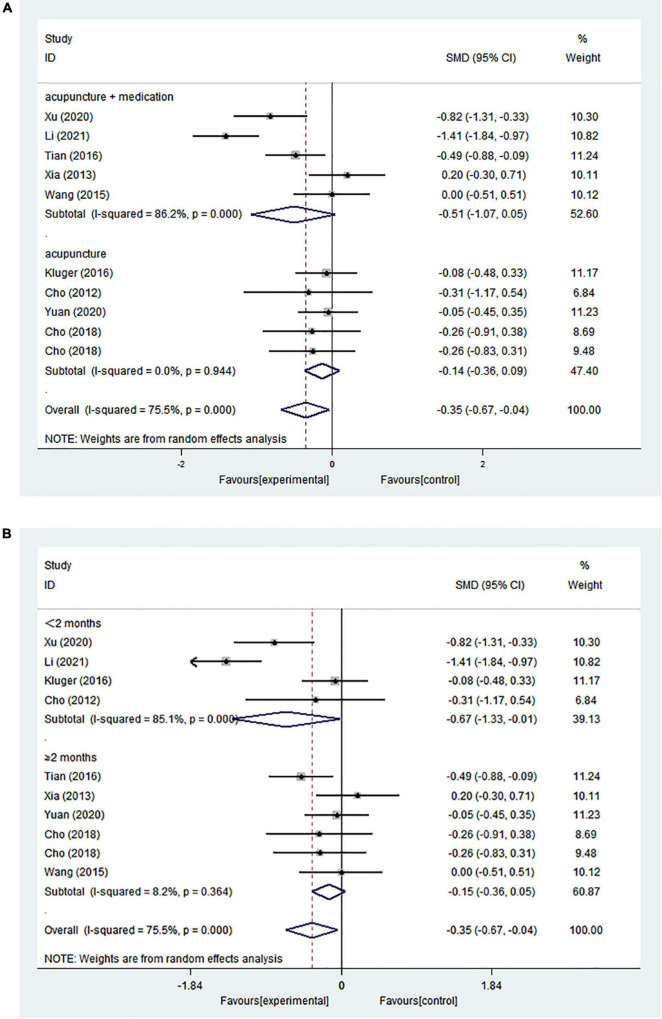
**(A)** Forest plot of effects of acupuncture treatment on PD-related depression according to intervention types. **(B)** Forest plot of effects of acupuncture treatment on PD-related depression according to course.

The pooled results from ten comparisons showed that acupuncture treatment had an overall significant positive intervention effect (SMD = −0.353; 95% CI = −0.669 to −0.037; *p* = 0.029) (Figure 4).

A subgroup analysis was performed on the basis of the intervention treatment because of significant heterogeneity (*I*^2^ = 75.5%). Meta-analysis revealed high heterogeneity (*I*^2^ = 86.2%) in the acupuncture combined with medication groups but no heterogeneity in the acupuncture alone group (*I*^2^ = 0%). However, both subgroups showed no statistically significant differences in efficacy for improving depression (acupuncture combined with medication: SMD = −0.509; 95% CI = −1.067 to 0.049; *p* = 0.074) (acupuncture alone: SMD = −0.136; 95% CI = −0.364 to 0.092; *p* = 0.241) (Figure 4A).

We further performed a subgroup analysis based on the intervention course; studies were separated into those evaluating outcomes within 2 months and those with evaluations after no less than 2 months. Statistically significant heterogeneity (*I*^2^ = 85.1%) and a statistically significant advantage (SMD = −0.671; 95% CI = −1.332 to −0.011; *p* = 0.046) were demonstrated in the studies with courses within 2 months. However, the other subgroup had lower heterogeneity (*I*^2^ = 8.2%) and showed no statistically significant differences in treatment efficacy (SMD = −0.153; 95% CI = −0.360 to 0.053; *p* = 0.145) (Figure 4B). Subgroup analyses showed that the intervention treatment or intervention course explained part of the heterogeneity.

#### Cognition

**FIGURE 5:**
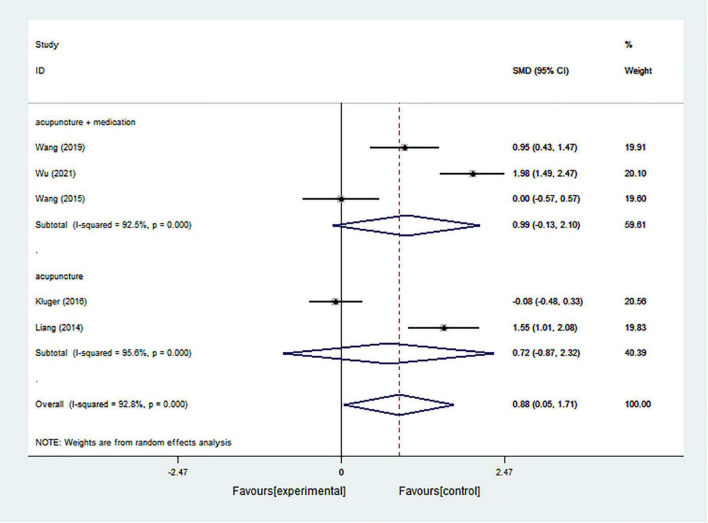
Forest plot of effects of acupuncture treatment on PD-related cognition according to intervention types.

The pooled results from 5 studies indicated that acupuncture had an overall significant positive intervention effect in improving cognition in PD patients (SMD = 0.878; 95% CI = 0.046–1.711; *p* = 0.039) with significant heterogeneity (*I*^2^ = 92.8%) (Figure 5). According to the subgroup analysis based on intervention types, both acupuncture alone and acupuncture combined with medication showed no statistically significant effect (acupuncture combined with medication: SMD = 0.985; 95% CI = −0.130 to 2.101; *p* = 0.083) (acupuncture alone: SMD = 0.724; 95% CI = −0.868 to 2.316; *p* = 0.373) with significant heterogeneity (*I*^2^ = 92.5% and *I*^2^ = 95.6%, respectively) (Figure 5).

#### Constipation

**FIGURE 6:**
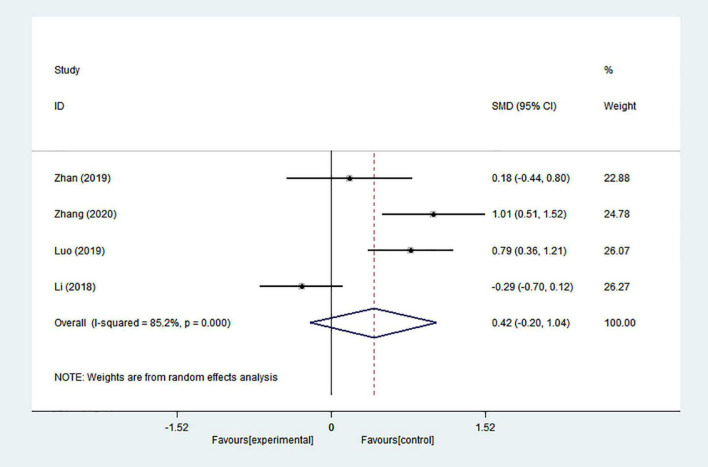
Forest plot of effects of acupuncture treatment on PD-related constipation.

Substantial heterogeneity was present in the comparison between acupuncture and medication in four studies (*I*^2^ = 65.5%). Statistically significant differences were not found in the pooled results (SMD = 0.422; 95% CI = −0.201 to 1.044; *p* = 0.185) (Figure 6).

#### Fatigue

Only one study provided information on fatigue, so a quantitative synthesis could not be performed. This trial included 94 PD patients who were randomized to receive 6 weeks of biweekly real or sham acupuncture. Both acupuncture and sham acupuncture showed significant improvements in the modified Fatigue Impact Scale scores, but no significant difference between the groups was found. The authors suggested that acupuncture improved PD-related fatigue through non-specific or placebo effects.

### Secondary outcomes

#### Unified Parkinson’s Disease Rating Scale I

**FIGURE 7:**
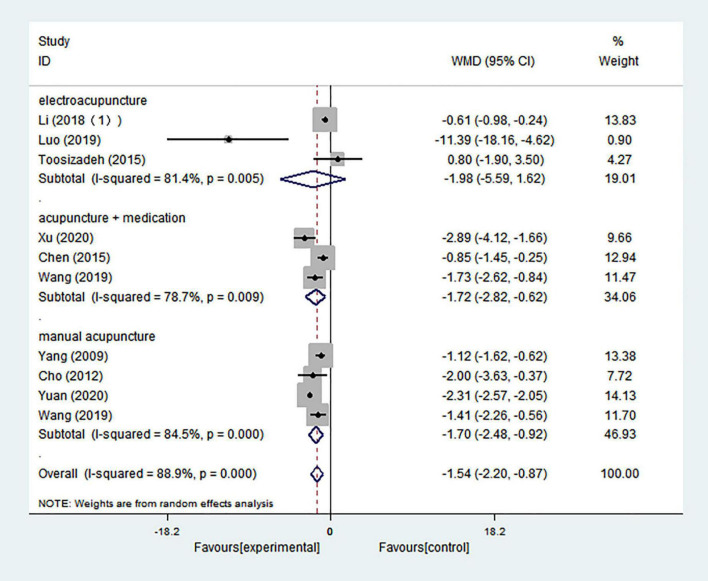
Forest plot of effects of acupuncture treatment on scores of UPDRS I according to intervention types.

In this analysis, 10 comparisons indicated that UPDRS I scores in PD patients could be significantly decreased by acupuncture treatment (WMD = −1.536; 95% CI = −2.201 to −0.871; *p* = 0.000), and there was high heterogeneity (*I*^2^ = 88.9%) (Figure 7). Intervention types were divided into manual acupuncture, electroacupuncture, and acupuncture combined with medication. Both manual acupuncture and acupuncture combined with medication performed significantly better in decreasing UPDRS I scores than the control treatment (WMD = −1.697; 95% CI = −2.476 to −0.918; *p* = 0.000; WMD = −1.718; 95% CI = −2.820 to −0.616; *p* = 0.002; respectively), and there was high heterogeneity (*I*^2^ = 84.5% and *I*^2^ = 78.7%, respectively). However, three studies evaluating an electroacupuncture intervention showed no significant effect (WMD = −1.984; 95% CI = −5.586 to 1.619; *p* = 0.280), and there was high heterogeneity (*I*^2^ = 81.4%) (Figure 7).

#### Unified Parkinson’s Disease Rating Scale II

**FIGURE 8:**
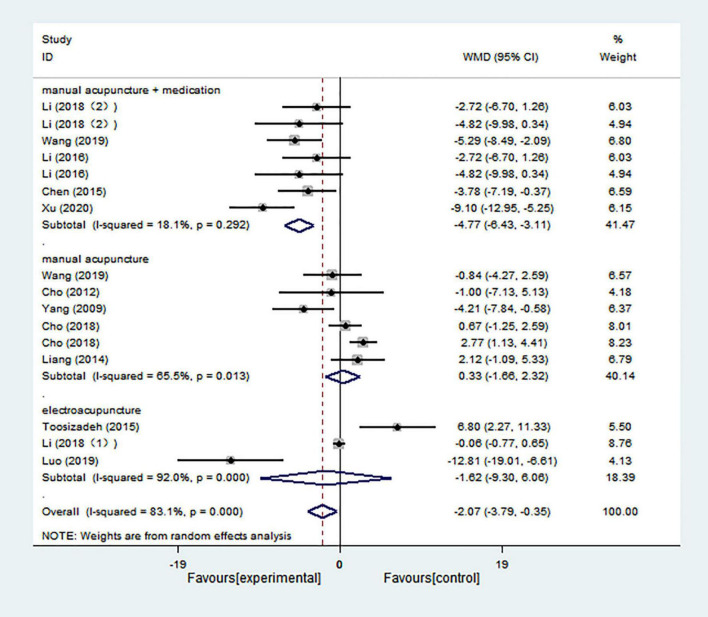
Forest plot of effects of acupuncture treatment on scores of UPDRS II according to intervention types.

The overall results based on 16 comparisons showed that acupuncture treatment had a better effect in decreasing UPDRS II scores than control treatment (WMD = −2.071; 95% CI = −3.792 to −0.351; *p* = 0.018), and there was high heterogeneity (*I*^2^ = 83.1%) (Figure 8). Both manual acupuncture and electroacupuncture had no significant effect in decreasing UPDRS II scores compared with control treatment (WMD = 0.333; 95% CI = −1.659 to 2.324; *p* = 0.743; WMD = −1.618; 95% CI = −9.301 to 6.065; *p* = 0.680, respectively), and there was high heterogeneity (*I*^2^ = 65.5% and *I*^2^ = 92.0%, respectively). Only manual acupuncture combined with medication was significantly better in decreasing UPDRS II scores (WMD = −4.768; 95% CI = −6.426 to −3.110; *p* = 0.000), and there was low heterogeneity (*I*^2^ = 18.1%) (Figure 8).

#### Quality of life

**FIGURE 9:**
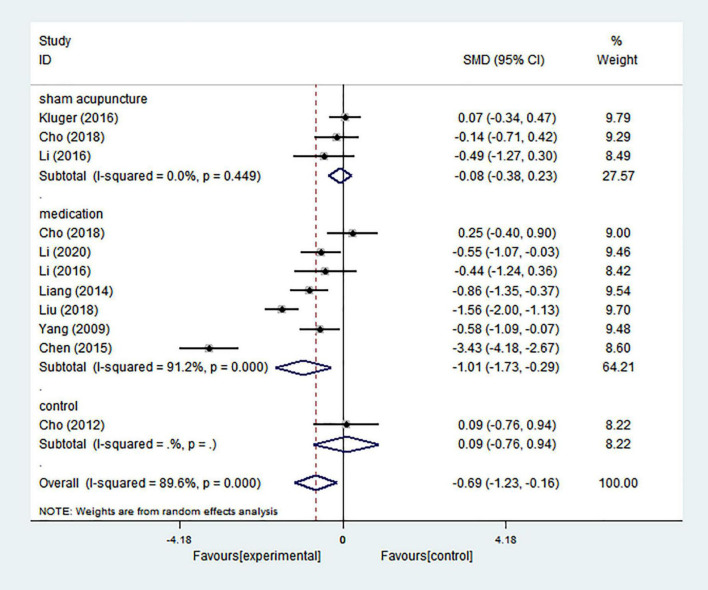
Forest plot of effects of acupuncture treatment on PD-related quality of life according to control types.

Pooling the results of 11 comparisons showed that acupuncture treatment could improve quality of life in patients with PD (SMD = −0.690; 95% CI = −1.226 to −0.155; *p* = 0.011), and there was high heterogeneity (*I*^2^ = 89.6%) (Figure 9). Based on the differences in control groups, we carried out a subgroup analysis (Figure 9). Meta-analysis of three comparisons showed no significant improvement in quality of life compared with sham acupuncture (SMD = −0.076; 95% CI = −0.380 to 0.227; *p* = 0.622), and there was no heterogeneity (*I*^2^ = 0.00%). Seven comparisons with medication indicated that quality of life could be improved (SMD = −1.013; 95% CI = −1.734 to −0.292; *p* = 0.006), and there was high heterogeneity (*I*^2^ = 91.2%). Only one comparison showed that there was no significant difference in improving quality of life between acupuncture alone and no treatment (SMD = 0.089; 95% CI = −0.761 to 0.939; *p* = 0.837). It was found that heterogeneity greatly decreased when the control group subgroup analysis was performed, which demonstrated that this may be one of the reasons for high heterogeneity.

### Sensitive analysis

A sensitivity analysis was conducted by excluding one study at a time, and this exclusion of studies one by one demonstrated that the results were not robust. Stable effects were present in the pooled results regarding quality of life and UPDRS I scores, while other pooled results, including PD-related insomnia, depression, cognition, and constipation were not stable. More details are shown in Supplementary Figures 1–7.

### Publication bias

Egger’s test based on different primary outcomes showed no significant publication bias of the included studies (insomnia outcome: Egger’s test *p* = 0.053; depression outcome: Egger’s test *p* = 0.816; cognition: Egger’s test *p* = 0.538; constipation: Egger’s test *p* = 0.793; UPDRS I: Egger’s test *p* = 0.918; UPDRS II: Egger’s test *p* = 0.067; and quality of life: Egger’s test *p* = 0.783).

## Discussion

### Summary of findings

Medication treatment is the main treatment for PD, but its long-term use may increase potential complications. Dopaminergic therapies have some limitations regarding treatment of the non-motor symptoms of PD (Muhammed et al., 2016). Adverse effects should also be considered alongside benefits when these drugs are prescribed, and alternative treatments should certainly be explored. In this study, we included 27 trials to evaluate whether acupuncture treatment is an effective option for treating the non-motor symptoms of PD.

In terms of PD-related insomnia, the subgroup results by type of intervention indicated that only acupuncture combined with medication but not acupuncture alone showed benefits. In some studies, it was found that acupuncture combined with medication was more effective, which was consistent with our findings (Cao et al., 2009, 2019; Dong et al., 2017; Zhao et al., 2021). In addition, subgroup analysis based on the intervention course indicated that 8 weeks may be the optimal course for improving PD-related insomnia. Moreover, some studies suggested that acupuncture may more effectively improve insomnia at 8 weeks (Garland et al., 2019). In addition, acupuncture has been shown to be effective for various types of insomnia, including primary insomnia, insomnia after stroke, depression-related insomnia, and chronic pain-related insomnia (Lee and Lim, 2016; Dong et al., 2017; Yin et al., 2017; Liu et al., 2019). It seemed that acupuncture worked on this symptom but not only PD-related insomnia specific, which still needed to be further studied and discussed.

Depression is a common feature in PD, with an estimated prevalence of 30–50% of individuals (Goodarzi et al., 2016). Our findings demonstrated that acupuncture treatment compared with the control treatments resulted in an overall statistically significant improvement in depression. This result was consistent with some previous studies (Xia et al., 2012; Liu et al., 2017; Subramanian, 2017). A previous review focusing on PD-related depression conducted a qualitative analysis of three trials and suggested that acupuncture had a positive effect on PD-related depression (Subramanian, 2017), which was consistent with our findings. Subgroup analysis based on the course showed that an intervention course within 2 months may be the optimal course for improving PD-related depression. Some associations were found in previous studies that demonstrated that the onset of acupuncture treatment effects occurred within 2 months (Andreescu et al., 2011; Wang Z. et al., 2017; Zhao et al., 2019). Although acupuncture could effectively ameliorate PD-related depressive symptoms, more evidence is needed because the sensitivity analysis was unstable to an extent. The unstable results might be ascribed to the stimulation parameters and duration of acupuncture in Xu and Tian’s studies. Besides, acupuncture treatment also had positive effect on patients with depression but without PD (Zeng et al., 2018; Li et al., 2019; Tu et al., 2021).

In terms of cognition, we found evidence of an overall greater effect of acupuncture treatment. A few studies focusing on PD-related cognition showed results consistent with this finding (Arankalle and Nair, 2013; Yeo et al., 2018). Other studies also indicated that acupuncture exerts a positive effect in improving global cognitive function, in conditions involving vascular cognition impairment, mild cognitive impairment, and Alzheimer’s disease-related cognitive impairment (He et al., 2021; Su et al., 2021; Zheng et al., 2021).

Constipation is one of the predominant early non-motor manifestations in PD, and there is pathological evidence to support the hypothesis that PD starts in the gut (Klingelhoefer and Reichmann, 2015). Our pooled results regarding constipation did not show the effect of acupuncture for PD-related constipation, which contradicted a previous study (Cheng, 2017). This result should be examined with caution due to the significant heterogeneity and small sample size. In fact, acupuncture was considered an effective or safe treatment for constipation in those without PD in some randomized trials (Liu et al., 2016; Lv et al., 2017; Wang B. et al., 2017). It is necessary to fill this gap by conducting high-quality evidence-based studies to confirm the effectiveness of acupuncture on constipation, especially on PD-related constipation.

Approximately 50% of PD patients report fatigue (Elbers et al., 2015). Among the studies included in this analysis, only one trial reported PD-related fatigue. This trial recruited 94 patients and suggested that acupuncture treatment may have a placebo effect on PD-related fatigue. In fact, holistic approaches (including acupuncture) are often advised for the treatment of fatigue (Dantzer et al., 2014). Acupuncture treatment had been showed in cancer-related fatigue, chronic fatigue syndrome (Zhang et al., 2018, 2019; Yin et al., 2021). In the absence of enough data allowing a quantitative analysis, the effect of acupuncture on PD-related fatigue remains uncertain based on the present evidence (Elbers et al., 2016).

Our results showed that manual acupuncture combined with medication or acupuncture alone might significantly decrease UPDRS I and II scores, which indicated that acupuncture could totally mitigate the non-motor symptoms of PD. The positive results from previous meta-analyses suggested the efficacy of acupuncture in reducing UPDRS I and II scores (Zhang et al., 2015; Noh et al., 2017). In terms of quality of life, our findings demonstrated that acupuncture treatment had statistically significant overall improvements. Consistent with findings of previous studies, acupuncture was associated with significant reductions in PDQ39 scores (Eng et al., 2006; Arankalle and Nair, 2013; Subramanian, 2017). The results of a subgroup analysis based on comparator types were in line with the finding obtained by Doo et al. (2015) that suggested that acupuncture treatment was more effective than medication in regard to PD-related quality of life (Doo et al., 2015).

### Limitations and strengths

To our knowledge, this study is the first to comprehensively examine acupuncture for non-motor symptoms of PD, although some symptoms (such as dizziness, bladder urgency, and apathy) were not reviewed because of the lack of original data. This study combined data from 27 eligible studies that covered an array of common non-motor symptoms and might provide potential evidence for one of non-pharmaceutical therapies for PD-related non-motor symptoms. Previous studies have mostly focused on motor symptoms (Chou et al., 2015; Lee and Lim, 2017). Other studies reviewed the effects of complementary therapies, including acupuncture, for motor and non-motor symptoms but did not provide a quantitative analysis (Subramanian, 2017; Deuel and Seeberger, 2020). Therefore, our study represents a comprehensive assessment of currently available evidence regarding PD non-motor symptoms.

As acupuncture is a manipulated intervention and an invasive non-drug therapy, blinding for acupuncturists is hard to implement in most studies, even in the studies outside of PD (He et al., 2020; Wang et al., 2022). Some concerns risk of bias mostly existed in the domains of blinding in our study, particularly in participant and personnel blinding, which weakened the strength of the evidence and our conclusions. Potential bias may be produced and placebo effect in acupuncture may confound these analyses. In an analysis of acupuncture for depression, it showed that acupuncture treatment had placebo effect (Smith et al., 2018). However, placebo effect of acupuncture treatment was conflicting in other studies (Sunay et al., 2011; Zhang et al., 2019). And trials using sham acupuncture may underestimate acupuncture’s treatment effect in some cases (Fei et al., 2022). Moreover, some of the pooled results continued to exhibit high levels of heterogeneity and were unstable in the sensitivity analysis, although we restricted the criteria for study enrollment and performed subgroup analyses. And most of the trials were conducted in China is one of the major limitations, which may affect the generalizability of the findings. Therefore, the effectiveness of acupuncture in PD-related non-motor symptoms should be interpreted with caution.

### Implications for clinical practice

Some results of this study may help provide guidance for clinical application. First, for patients with PD-related depression or decreased quality of life or cognition, acupuncture treatment may be a good choice for health care workers. Second, among acupuncture treatment types, acupuncture combined with medications may be more suitable for PD-related insomnia, and manual acupuncture combined with medication could be recommended for improving total mentation, behavior and mood (UPDRS I) or activities of daily living (UPDRS II). Third, 8 weeks may be the optimal course for improving PD-related insomnia and within 2 months for PD-related depression.

## Conclusion

In conclusion, this meta-analysis demonstrated that acupuncture treatment may be associated with improvements in PD-related depression, quality of life, cognition, total mentation, behavior and mood, and activities of daily living. However, more evidence, especially a higher level of evidence from large-scale clinical trials, is still needed to support these clinical recommendations.

## Data availability statement

The original contributions presented in this study are included in the article/Supplementary material, further inquiries can be directed to the corresponding author.

## Author contributions

CW and MZ designed this study, supervised the research, and reviewed the manuscript. XW, ML, LZ, and ZL performed the articles screening and data extracting. QL and XH performed the quality assessment. QL contributed to data analysis, and wrote and reviewed the manuscript. XW, LZ, and ZL edited the figures and table. All authors contributed to the article and approved the submitted version.
