# Supplementary material for: Effect of acupuncture for non-motor symptoms in patients with Parkinson’s disease: A systematic review and meta-analysis
Source: Front Aging Neurosci. 2022 Oct 6;14:995850. doi: 10.3389/fnagi.2022.995850 (PMC9582755; doi:10.3389/fnagi.2022.995850)
Supplement: Supplementary file 1 [file Data_Sheet_1.doc]

## Search strategy

**MEDLINE(OVID) (counts:173)**

1. Parkinson.tw.
2. Parkinson$.tw.
3. (PD or IPD).tw.
4. (Parkinson$ adj5 Diseas$).tw.
5. exp Parkinson Disease/

6. or/1-5

7. exp Acupuncture Therapy/

8.(acupunct* or electroacupunct* or electro-acupunct* or meridian* or acupoint* or acupuncture points or body acupunct* or fire needl* or warm needl* or intradermal needl* or scalp needl* or scalp acupunct* or ear needl* or auricular needl* or auricular acupunct* or ear acupunct* or float needl* or abdominal needl* or abdominal acupunct* or eye needl* or wrist-ankle acupunct* or dry needl* or elongated needl*).mp.

9.or/7-8

10. randomized controlled trial.pt.

11. controlled clinical trial.pt.

12. randomized.ab.

13. placebo.ab.

14. randomly.ab.

15. trial.ab.

16. groups.ab.

17. or/ 10-16

18. 6 and 9 and 17

**Embase(OVID) (counts: 244)**

1. Parkinson.tw.
2. Parkinson$.tw.

3 (PD or IPD).tw.

4 (Parkinson$ adj5 Diseas$).tw.

5 exp Parkinson Disease/

6 1 or 2 or 3 or 4 or 5

7. randomized controlled trial.pt.

8. controlled clinical trial.pt.

9. randomized.ab.

10. placebo.ab.

11. randomly.ab.

12. trial.ab.

13. groups.ab.

14. or/7-13

15. exp acupuncture/

16. (acupunct* or electroacupunct* or electro-acupunct* or meridian* or acupoint* or acupuncture points or body acupunct* or fire needl* or warm needl* or intradermal needl* or scalp needl* or scalp acupunct* or ear needl* or auricular needl* or auricular acupunct* or ear acupunct* or float needl* or abdominal needl* or eye needl* or wrist-ankle acupunct* or dry needl* or elongated needl*).mp.

17. or/15-16

19. 6 and 14 and 17

**Cochrane library (counts: 226)**

#1 Parkinson Disorders

#2 parkinson*

#3 PD or IPD

#4 #1 or #2 or #3

#5 acupunct* or electroacupunct* or electro-acupunct* or meridian* or acupoint* or acupuncture points or body acupunct* or fire needl* or warm needl* or intradermal needl* or scalp needl* or scalp acupunct* or ear needl* or auricular needl* or auricular acupunct* or ear acupunct* or float needl* or abdominal needl* or eye needl* or wrist-ankle acupunct* or dry needl* or elongated needl*

#6 #4 and #5

Trials

**Web of science（counts: 75）**

Indexes=SCI-EXPANDED, SSCI, A&HCI, ESCI, Timespan=All years

#1 TS=(Parkinson*) OR TI=(Parkinson*)

#2 TS=(trial) OR TI=(trial) OR TI=(random*) OR TI=(placebo) OR TI=(control*)

#3 TS=(acupunct*) OR TS=(electroacupunct*) OR TS=(electro-acupunct*) OR TS=(meridian*) OR TS=(acupoint*) OR TS=(acupuncture points) OR TS=(body acupunct*) OR TS=(fire needl*) OR TS=(warm needl*) OR TS=( intradermal needl*) OR TS=(scalp needl*) OR TS=(scalp acupunct*) OR TS=(ear needl*) OR TS=(auricular needl*) OR TS=(auricular acupunct*) OR TS=(ear acupunct*) OR TS=(float needl*) OR TS=(abdominal needl*) OR TS=(eye needl*) OR TS=(wrist-ankle acupunct*) OR TS=(dry needl*) OR TS=(elongated needl*)

#4 #1 AND #2 AND #3

**Chinese database**

**China National Knowledge Infrastructure (CNKI)** **(counts: 487）**

( SU='针灸' OR SU='针刺' OR SU='电针' OR SU='头针' OR SU='耳针' OR SU='腹针' OR SU='浮针' OR SU='眼针' OR SU='皮内针' OR SU='火针' OR SU='温针' OR SU='体针' OR SU='腕踝针' OR SU='干针' OR SU='芒针' ) AND (SU='帕金森' OR SU='帕金森病') AND (TKA='随机' OR TKA='对照')

**Chinese BioMedical Literature Database (CBM) (counts: 423)**

(针刺 or 针灸 or 电针 or 头针 or 耳针 or 腹针 or 浮针 or 眼针 or 皮内针 or 火针 or 温针or 体针 or 腕踝针 or 干针 or 芒针) and (帕金森 or 帕金森病) and (随机 or 对照)

**Wangfang Database (counts: 760)**

主题=(针刺 or 针灸 or 电针 or 头针 or 耳针 or 腹针 or 浮针 or 眼针 or 皮内针 or 火针 or 温针or 体针 or 腕踝针 or 干针 or 芒针) and 主题=(帕金森 or 帕金森病) and 主题=(随机 or 对照 )

**Chonqing VIP (CQVIP) (counts: 24)**

U=(针刺 or 针灸 or 电针 or 头针 or 耳针 or 腹针 or 浮针 or 眼针 or 皮内针 or 火针 or 温针or 体针 or 腕踝针 or 干针 or 芒针) AND U=(帕金森 or 帕金森病) AND U=(随机 or 对照 )

**Supplementary Figure**

Supplementary Figure 1.


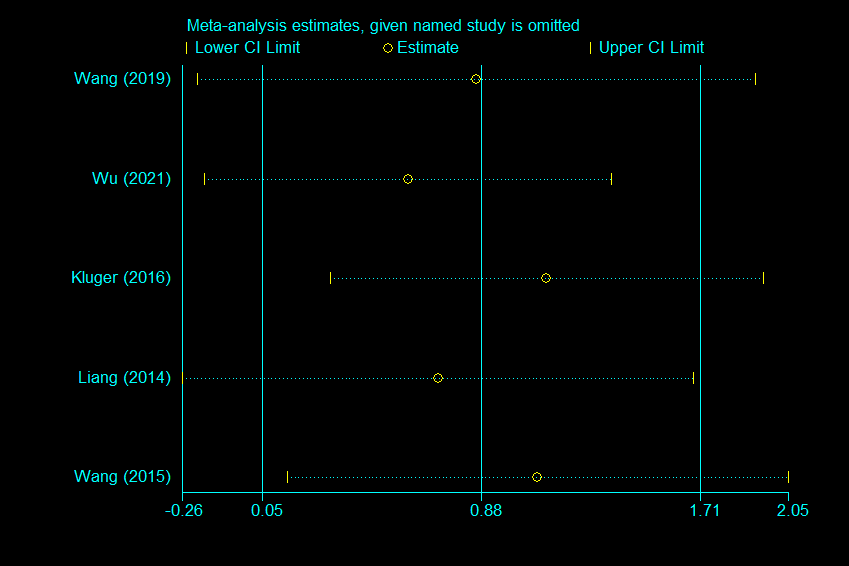


Supplementary cognition sensitive analysis

Supplementary Figure 2.


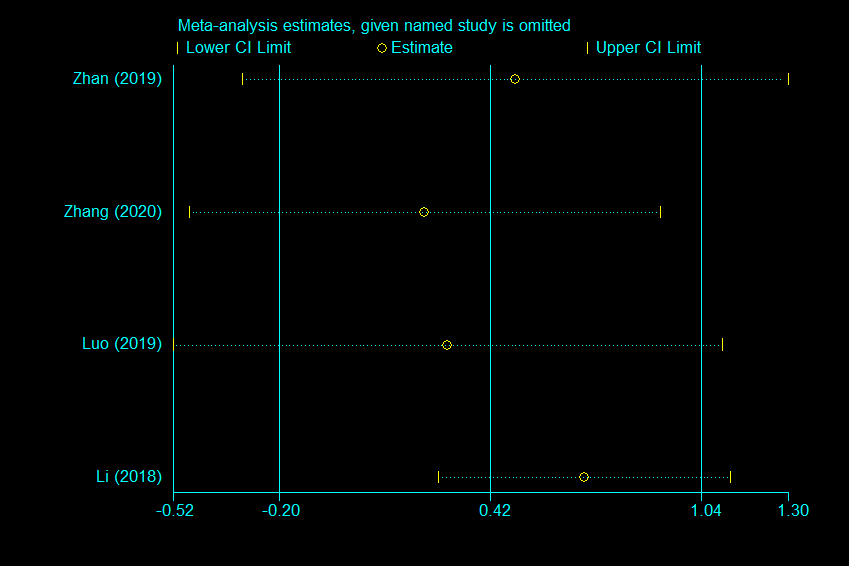


Supplementary constipation sensitive analysis

Supplementary Figure 3.


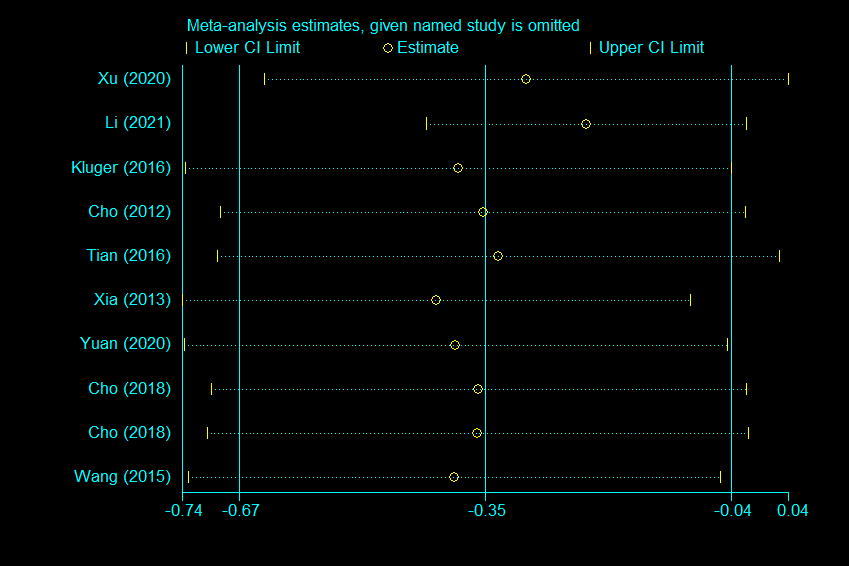


Supplementary depression sensitive analysis

Supplementary Figure 4.


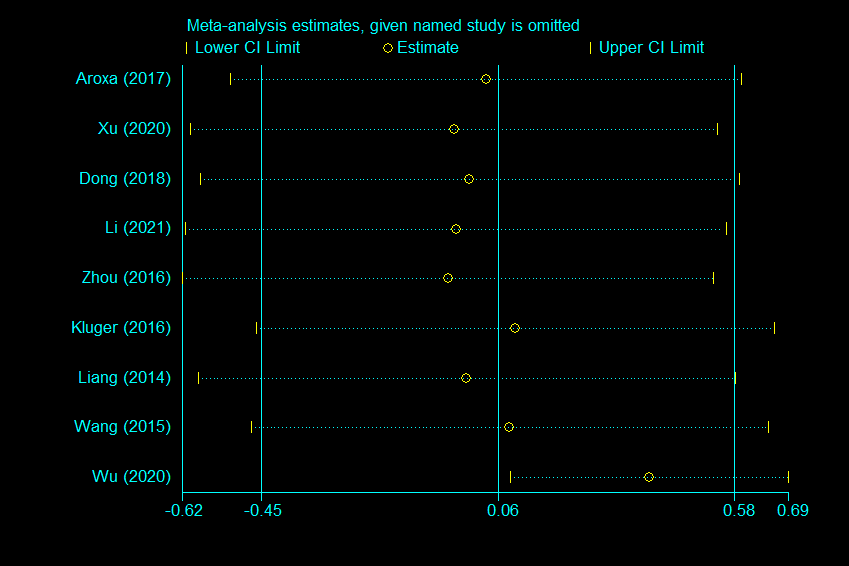


Supplementary insomnia sensitive analysis

Supplementary Figure 5.


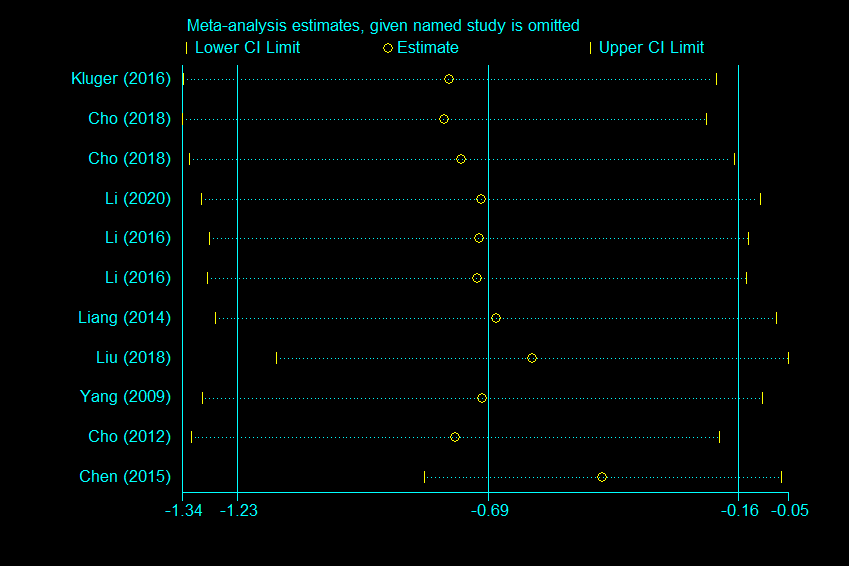


Supplementary quality of life sensitive analysis

Supplementary Figure 6.


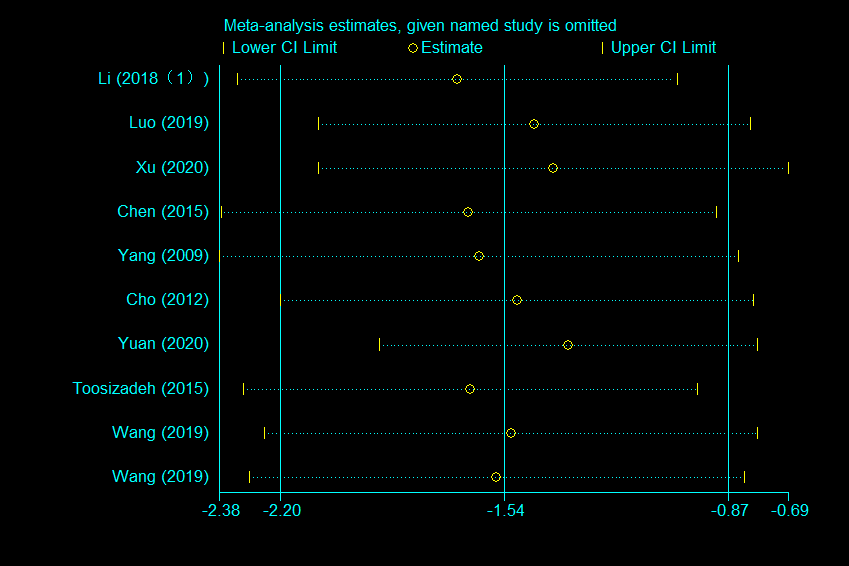


Supplementary UPDRSⅠsensitive analysis

Supplementary Figure 7.


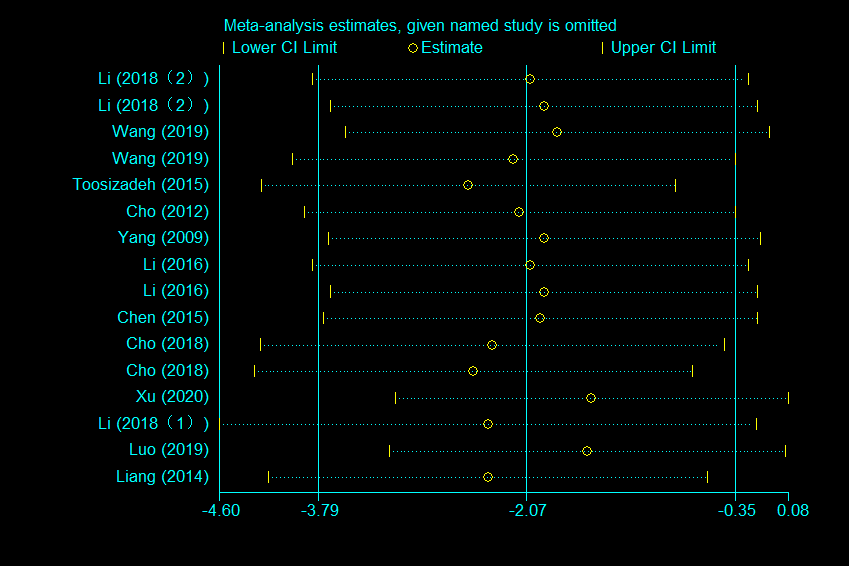


Supplementary UPDRSⅡsensitive analysis
